# At the Crossroads of Continents: Ancient DNA Insights into the Maternal and Paternal Population History of Croatia

**DOI:** 10.3390/genes17010080

**Published:** 2026-01-09

**Authors:** Damir Marjanović, Jelena Šarac, Dubravka Havaš Auguštin, Mario Novak, Željana Bašić, Ivana Kružić, Natalija Novokmet, Olivia Cheronet, Pere Gelabert, Ron Pinhasi, Gordan Lauc, Dragan Primorac

**Affiliations:** 1 Centre for Applied Bioanthropology, Institute for Anthropological Research, 10000 Zagreb, Croatia; dmarjanovic@inantro.hr (D.M.); mnovak@inantro.hr (M.N.); natalija.novokmet@inantro.hr (N.N.); 2 Department of Genetics and Bioengineering, International Burch University, 71000 Sarajevo, Bosnia and Herzegovina; 3 Faculty of Biotechnology and Drug Development, University of Rijeka, 51000 Rijeka, Croatia; 4 Department of Archaeology and Heritage, Faculty of Humanities, University of Primorska, 6000 Koper, Slovenia; 5 Faculty of Forensic Sciences, University of Split, 21000 Split, Croatia; zbasic@forenzika.unist.hr (Ž.B.); ianteric@forenzika.unist.hr (I.K.); 6 Department of Evolutionary Anthropology, University of Vienna, 1030 Vienna, Austria; olivia.cheronet@univie.ac.at (O.C.); pere.gelabert@univie.ac.at (P.G.); ron.pinhasi@univie.ac.at (R.P.); 7 Human Evolution and Archaeological Sciences (HEAS), University of Vienna, 1030 Vienna, Austria; 8 Genos Glycoscience Research Laboratory, 10000 Zagreb, Croatia; glauc@genos.hr; 9 Faculty of Pharmacy and Biochemistry, University of Zagreb, 10000 Zagreb, Croatia; 10 St. Catherine Specialty Hospital, 10000 Zagreb, Croatia; draganprimorac2@gmail.com; 11 Faculty of Dental Medicine and Health, Josip Juraj Strossmayer University of Osijek, 31000 Osijek, Croatia; 12 School of Medicine, Josip Juraj Strossmayer University of Osijek, 31000 Osijek, Croatia; 13 Medical School, University of Split, 21000 Split, Croatia; 14 Department of Biochemistry & Molecular Biology, The Pennsylvania State University, State College, PA 16802, USA; 15 The Henry C. Lee College of Criminal Justice and Forensic Sciences, University of New Haven, West Haven, CT 06516, USA; 16 Regiomed Kliniken, 96450 Coburg, Germany; 17 Medical School, University of Rijeka, 51000 Rijeka, Croatia; 18 Gandhinagar Campus, National Forensic Sciences University, Gandhinagar 382007, India

**Keywords:** mtDNA, Y chromosome, ancient DNA, Croatian (pre)history

## Abstract

Background/Objectives: Southeastern Europe and Croatia have served as a genetic crossroads between the Near East and Europe since prehistoric times, shaped by numerous and repeated migrations. By integrating 19 newly generated ancient genomes with 285 previously published ancient genomes from Croatia, we investigated patterns of maternal and paternal landscapes from the Neolithic, Bronze, and Iron Ages through to the Antiquity and medieval periods, as well as the modern Croatian population. Methods: Ancient DNA extraction from human remains and library preparation were conducted in dedicated clean-room facilities, followed by high-throughput sequencing on the Illumina platform. Sequencing data were analyzed with established pipelines to determine mitochondrial and Y-chromosomal haplogroups and the genetic sex of individuals. Results: New ancient data reveal a predominantly European maternal profile, dominated by haplogroups H, U, and HV0, whereas Y-chromosomal lineages are characterized by J subclades and R1a, with limited representation of R1b and the absence of I2a. When combined with published ancient Croatian genomes, the results reveal similar haplogroup diversity and patterns, as well as the expansion of mtDNA haplogroup H over time and a substantial increase in Y-chromosome R1a and I2a haplogroup frequency from the prehistoric to the modern period. Conclusions: Although the analyzed samples are heterogeneous and originate from different historical periods, their genetic signatures conform to the broader patterns expected for the region. In a wider context, the ancient Croatian mitochondrial data reveal stronger genetic persistence from prehistory to modern times, unlike paternal lineages, which show significantly higher divergence.

## 1. Introduction

Over the past few decades, molecular anthropology (the analysis of uniparental genetic markers, such as mitochondrial DNA and the Y chromosome, alongside genome-wide data) has profoundly transformed reconstructions of human population history, providing increasingly detailed insights into past demographic processes. These approaches have been particularly informative in regions characterized by long-term population interaction and mobility, where successive cultural and genetic influences have accumulated over a long time period. One of these regions is Southeastern Europe (SEE), including present-day Croatia, which occupies a key position at the crossroads of the Carpathian Basin, the Balkans, and the eastern Adriatic and has served as a genetic and cultural corridor linking the Near East, the Aegean, and the Pontic–Caspian steppe for millennia [1,2,3,4,5,6]. It was also among the first parts of Europe to be influenced by migrations from Anatolia, associated with the spread of farming during the early Neolithic period (around 6000 BCE) [7,8].

Previously published genetic analysis of the modern SEE gene pool confirmed the extraordinary heterogeneity and complexity of the migrations and population admixtures in this region, and the latest ancient DNA analyses have revealed interesting new insights in this context. Namely, ancient DNA data from the “Southern Arc” showed how migrations between West Asia, the Caucasus, and SEE shaped early Indo-European populations, with SEE serving as a genetic crossroads where steppe ancestry mixed with Anatolian and local farmer components [4]. Subsequent genome-wide studies of the historic Balkans revealed substantial Anatolian-related admixture during the Roman Imperial period, followed by renewed gene flow from Central and Eastern Europe during Late Antiquity and the early medieval Slavic migrations, which contributed up to 60% of modern SEE ancestry [5].

Mitochondrial DNA (mtDNA) diversity in SEE reflects a sequence of major demographic events spanning the Late Pleistocene to recent historical times. Mesolithic hunter-gatherers of the Balkans and the wider Danube Basin were predominantly characterized by haplogroups H and U (especially U5, U4, and U2), consistent with a broader European pattern [4,9]. From the paternal perspective, the dominant SEE genetic signature of Mesolithic hunter-gatherers is represented by Y-chromosome haplogroup I2a, which is consistently recovered from pre-Neolithic archeological contexts throughout the region and is suggested to have a Paleolithic origin in this region [2,10]. Mathieson et al. (2018) [9] demonstrate that Mesolithic individuals from the Danube Gorges (e.g., Lepenski Vir, Vlasac, and Padina) were carriers of I2a, highlighting a deep genetic persistence of this lineage in the Balkan Peninsula since the Late Upper Paleolithic.

The arrival of early Neolithic farmers from the Aegean and northwestern Anatolia in the 7th millennium BCE introduced a contrasting maternal profile enriched in haplogroups such as U, K1, N1a, and T, which spread through SEE and admixed with local foragers, as documented in Aegean/Anatolian Neolithic and early Balkan farming communities [9]. The paternal counterpart reflecting the Near Eastern contribution in SEE is represented primarily by Y-chromosome haplogroups G and J. Namely, Neolithic individuals from the Aegean, the Balkans, and Central Europe frequently carried G2a, the dominant Y-lineage among early Anatolian farmers, alongside emerging evidence for J2 and related subclades [9,11,12,13,14].

**Figure 1:**
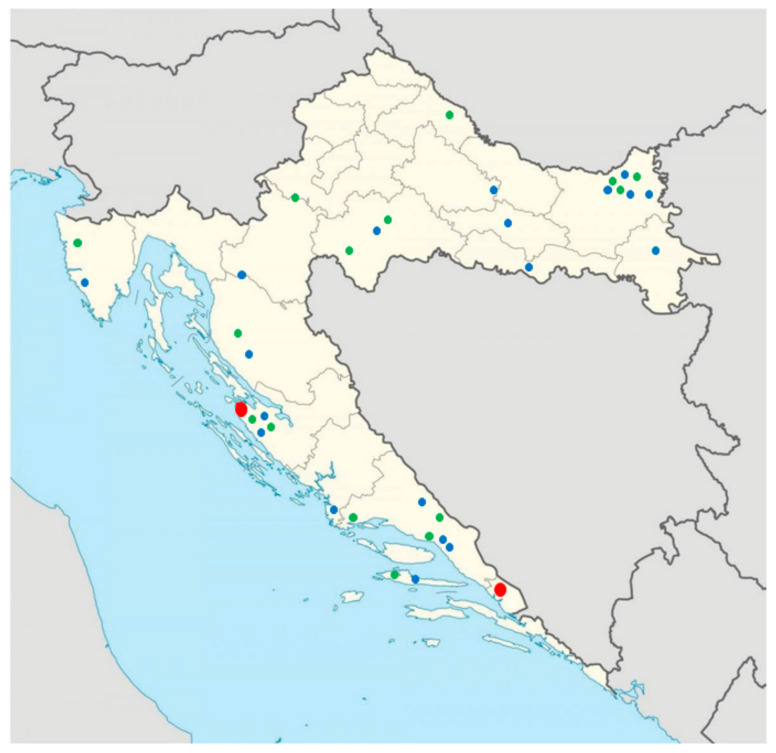
Map of Croatia with location of excavation sites of new ancient samples (red dots), prehistoric published samples (blue dots), and historic published samples (green dots).

During the subsequent Copper and Bronze Ages, large-scale migrations and gene flow from the Pontic–Caspian steppe and later Near Eastern sources added new layers to the genetic landscape. SEE was, at that period, dominated by mitochondrial haplogroups H, J, T, K, and U, as well as the Y-chromosome haplogroup R1b, followed by a medieval Slavic signature embodied in the Y haplogroup R1a and southern lineages such as E1b [4,10,15]. Although most previous aDNA research has focused on autosomal data and genome-wide variations, uniparental markers offer an interesting additional perspective due to their lineage-specific transmission, which makes them especially valuable for tracing population movements [6,16]. The aim of this study is therefore to investigate population continuity and changes in uniparental lineages over time in present-day Croatia by integrating 19 new mitochondrial and Y-chromosomal datasets from Dalmatia, Croatia, with 285 existing ancient genomes from different (pre)historic Croatian sites (Figure 1).

## 2. Materials and Methods

### 2.1. Description of New Sample and Locations

A total of 20 human remains (teeth) from the following archaeological sites were selected for genetic analysis: including Ljubač, nekropola Venac; Krneza, Duševića glavica; and Vrbica, Dračevac, and Torine, tumuli. Of these 19 samples yielded enough data for downstream genetic analyses (Figure 1, Supplementary Table S1). The excavated tumuli represent prehistoric mounds, likely of Bronze Age origin, later reused in Antiquity and the medieval period. Tumulus 2 contains a single prehistoric cist burial; tumuli 3 and 11 preserve only disturbed remains. Tumulus 20 exhibits an early Iron Age burial with Hellenistic and medieval intrusions, while tumulus 21 contains a prehistoric cist with an additional bone concentration [17]. The tumulus at Duševića glavica, excavated in 2008, is an earthen mound built over compacted stones that contains eleven graves, including two prehistoric burials and nine early medieval burials [18]. The Venac necropolis at Ljubač, excavated in 2009–2010, consists of 35 graves organized into older mound burials dated to the late 10th–7th century BC and younger flat graves from the 7th–6th centuries BC, forming a coherent unit with the nearby hillfort [19]. The broader tumulus zone on Ljubačka kosa contains a dense concentration of prehistoric mounds. Despite earlier disturbances, excavations have revealed Late Bronze and Early Iron Age material, reflecting long-term funerary use connected to the Venac hillfort [19].

### 2.2. Bioarcheological Analysis of Skeletal Remains

The osteological material recovered from the site was further analyzed at the Faculty of Forensic Sciences, University of Split, Croatia. Each individual’s biological profile was estimated following standard procedures. Age at death was estimated from changes in the pubic symphysis [20,21], supplemented by observations of degenerative joint changes [22] and dental wear patterns [23]. Each skeleton was examined macroscopically for pathological alterations and traumatic lesions [24,25,26,27].

### 2.3. DNA Isolation, Library Preparation, and Sequencing

All ancient DNA (aDNA) analyses were performed in dedicated clean-room facilities at the Ancient DNA Laboratory, Department of Evolutionary Anthropology, Vienna, following strict anti-contamination protocols. Before the isolation of DNA, samples were mechanically cleaned by the sandblasting method to remove the outer surface and subsequently UV-irradiated to eliminate any exogenous DNA. DNA was extracted from 50 mg of material using the protocol described by Dabney et al. (2013) with adaptations from Korlevic et al. (2015) [28,29]. Double-stranded Illumina sequencing libraries were prepared from 12.5 µL of DNA extract following established ancient DNA protocols [30]. All reagents and consumables were UV-irradiated before use, and one negative control containing only reagents without template DNA was included for every batch of samples at each laboratory stage. Library preparation involved blunt-end repair, adapter ligation, and purification using the MinElute PCR Purification Kit (Qiagen, Hilden, Germany), with elution in 25 µL of EBT buffer (1 mM EDTA, 0.05% Tween-20). Indexed amplification was performed using the NEBnext Q5U enzyme from NEB (Ipswich, MA, USA) in a dedicated post-PCR facility, followed by final purification with 1.2 × NGS clean-up magnetic beads (Macherey-Nagel, Düren, Germany) per sample, introducing a size selection. We eluted the samples in 25 µL EBT buffer (1 mM EDTA, 0.05% Tween-20). These libraries were subjected to both shotgun and capture sequencing on the Illumina NovaSeqX platform using the 10B XP SR 100 workflow at the NGS facility of the Vienna BioCenter Core Facility (VBCF, Vienna, Austria). The capture was performed after an initial round of shallow screening sequencing. The libraries were enriched for human genomic DNA using the 1.4M ancient DNA panel from Twist. Pre- and post-capture amplifications were performed using the KAPA HiFi HotStart DNA Polymerase (Roche, Basel, Switzerland) and IS5/IS6 primer pair.

### 2.4. Bioinformatics and Data Analysis

Adapter sequences were trimmed from reads using Cutadapt (version 1.15) [30], discarding reads under 30 bp (−m 30) and allowing an overlap of 1 bp between the read and adapter (−O 1). Trimmed reads were collapsed using PEAR 3.13.0 [31] using an overlap of 11 bases and a minimum length of 30 bp. Collapsed reads were inspected for problems using FASTQC v0.12.1 [32]. These were aligned against the human reference hg19 (GRCh37), the revised Cambridge Reference Sequence (rCRS, Genbank accession no. NC_012920.1) by using BWA aln (version 0.7.15-r1140), disabling seeding, and setting a gap penalty open to 2 and an edit distance of 0.01. 

We assessed aDNA authenticity using several criteria: a cytosine deamination rate above 15%, a read length distribution compatible with aDNA, and an upper-bound rate for the consensus mitochondrial sequence above 95% using contamMix-1.0.1 [33]. Samples showing contamination were tagged and discarded if two contamination signals were present.

Coverage on the autosomal and sex chromosomes was calculated using a script available at https://github.com/TCLamnidis/Sex.DetERRmine (accessed on 7 January 2025) to determine the genetic sex of each individual with standard errors. The presence of deamination damage patterns at the terminal bases of reads, characteristic of ancient DNA, was verified using mapDamage (version 2.0.8).

Schmutzi v.1.5.7 [34] was used to reconstruct consensus mtDNA sequences for each individual from the remapped reads, which were then imported into Haplogrep2 (https://haplogrep.i-med.ac.at/ (accessed on 7 January 2025)) [35] for automated mitochondrial haplogroup assignment based on phylotree mtDNA tree build 17 (http://www.phylotree.org/ (accessed on 7 January 2025)) [36]. Yleaf (version 1.0) was used to infer the Y-chromosomal haplogroup in males in an automated way based on haplogroup-defining SNP positions in the ISOGG 2016 nomenclature [37].

## 3. Results

### 3.1. Bioarcheological Analysis

Supplementary Table S1 summarizes the bioarcheological analysis, dating, pathologies, and grave findings. Skeletal preservation varied widely, allowing only partial reconstruction of biological and pathological profiles. Dental caries were common, with one case of dental abscess. Single instances of enamel hypoplasia and orbital cribra indicate childhood physiological stress. One adult showed habitual tooth-use wear. A few individuals exhibited age- or load-related degeneration, including mild to moderate osteoarthritis and one case of Schmorl’s nodes. Otitis media was noted in one individual, and no traumatic lesions were identified.

### 3.2. Genetic Analysis

A collection of 19 samples yielded sufficient endogenous DNA for genetic analysis, while 1 sample from Ljubač, nekropola Venac (Gr. 25) was too poorly preserved for downstream processing. Sex determination based on shotgun sequencing identified seven females, ten males, and three individuals of undetermined sex (Supplementary Table S1). Following capture sequencing, one previously unassigned individual was reclassified as male, resulting in a final dataset comprising 11 males, 7 females, and 2 individuals of undetermined sex based on nuclear genomic coverage.

#### 3.2.1. Mitochondrial DNA Haplogroup Composition of New Dalmatian Samples

**Figure 2:**
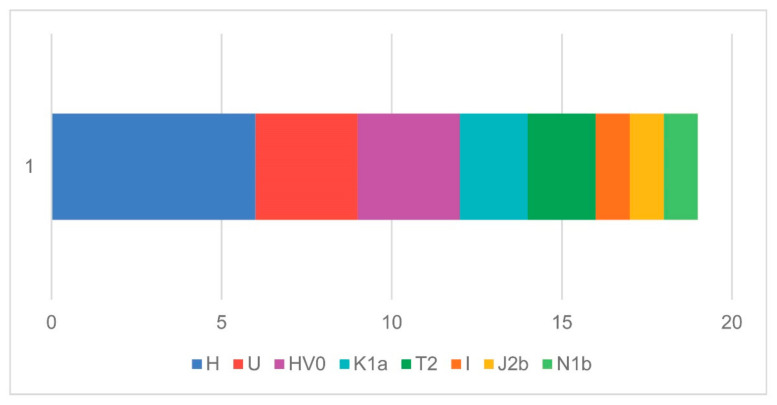
mtDNA haplogroup composition of new ancient Dalmatian samples.

Mitochondrial haplogroups show a composition typical of both (pre)historic and present-day European populations (Figure 2). No haplogroups indicative of non-European ancestry were detected. Although some deviations from modern frequencies exist, these are likely due to the small sample size and localized burial contexts. The most common haplogroup was H, detected in six individuals (31.6%) across three sites. Subclades H2, H2a, H2a2, H4d, and H5a1 were identified. Haplogroup H is the most frequent and diverse maternal lineage in Europe, present in approximately 40–50% of modern Europeans and widespread across North Africa and the Near East [38,39,40,41,42]. The second most frequent lineage was U, present in three individuals (15.8%), encompassing U2d2, U3a1, and U4a2. Haplogroup U represents one of Europe’s most ancient mitochondrial clades, dating to 45,000–55,000 years before the present [43,44]. While most U subclades are widespread in Europe, U2 and U3 appear less frequently and are more common in Near Eastern and South Asian populations [45,46], suggesting deep ancestral connections that extend beyond Europe.

Haplogroup HV0 was also observed in three individuals (15.8%). Given their shared archeological context (the same Ljubač, nekropola Venac site), kinship between these individuals cannot be excluded. However, HV0 occurs at low frequencies in modern Europe but is more common in the Near East [40,41], with ancient occurrences documented in Neolithic central Europe [47]. Haplogroups T2a, T2b, and J2b1c were identified in three individuals (15.8% combined), which is consistent with modern European averages [38,48]. Haplogroup K1a was present in two individuals (10%), reflecting continuity from the U8 lineage that originated in the Near East approximately 30,000 years ago [46]. Two rare haplogroups—I1a and N1b—were detected in single individuals (ca. 5% each). Haplogroup I occurs sporadically in western Europe, while N1b is exceedingly rare (usually below 2%) in modern European populations [49]. Overall, the maternal genetic landscape of ancient Dalmatian samples reflects genetic persistence with Neolithic and Bronze Age European populations, with haplogroups H, U, and HV0 dominating alongside minor but informative variation.

#### 3.2.2. Y-Chromosomal Haplogroup Composition of New Dalmatian Samples

**Figure 3:**
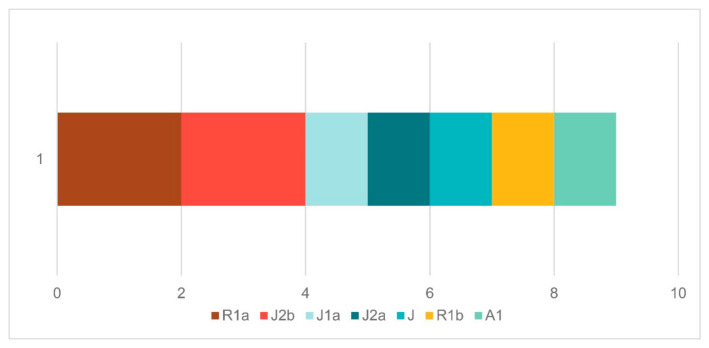
Y haplogroup composition of new ancient Dalmatian samples.

A total of 9 out of 11 male individuals gave sufficient information for Y-chromosomal haplogroup determination, encompassing seven distinct sublineages (Figure 3). The dominant paternal clades were J2b and R1a, each comprising roughly 22% of the male dataset. Together with additional J1a, J2a, and J* lineages (~55% combined), they suggest a strong genetic signature of Neolithic ancestry. Haplogroup J, which entered SEE during the Neolithic agricultural expansions, maintained a continuous presence through the Bronze Age and Antiquity, reflecting long-term genetic and cultural connections between the Adriatic, the Near East, and the wider Mediterranean [3,4,11]. The co-occurrence of J2a and J2b in the present dataset reinforces this scenario, as both are well-documented markers of Neolithic settlement and later regional interaction in SEE. R1a, today the second most common Y haplogroup in Croatia and SEE, was found in two individuals, one in the prehistoric part of the sample and one in the early historic part. Its presence confirms the occurrence of this haplogroup in prehistoric SEE. However, this is very scarce, and its rise in early medieval times is suggested to be linked with movements from Central/Eastern Europe, including those connected to Slavic expansions [2,5,6,10].

In contrast, the R1b component, reflecting the Bronze Age influx of steppe-related ancestry linked to the spread of Indo-European-speaking groups into Europe, appears in only a single prehistoric individual, even though its presence would be expected in more cases given that most of the samples date to the Bronze and Iron Ages [9,15,50]. Haplogroup I2a, the dominant paternal lineage in modern Croatian and SEE populations and previously suggested as a key marker of Paleolithic Balkan hunter-gatherers [2,10], was also absent in this dataset. This absence echoes earlier findings from early medieval Croatian and SEE samples, suggesting that I2a, although present in the region since Paleolithic times, may have risen to prominence through later demographic events [6].

Finally, one individual carried haplogroup A1 (ca. 11%), a lineage of deep African origin. Haplogroup A1 is extremely rare in both ancient and modern European datasets and cannot generally be linked to major Neolithic, Bronze Age, or early medieval population movements [51]. Its occurrence in this context may reflect episodic individual mobility or limited contact across the Mediterranean, rather than participation in broader demographic processes.

### 3.3. Integration of New Dalmatian Samples into a Database of Ancient Croatian Genomes

To gain a broader understanding of the maternal and paternal genetic landscape of Croatia during prehistory and history, we created a database comprising a total of 304 ancient DNA samples, which included 19 newly analyzed genomes and 285 previously published genomes. The dataset was also divided into 138 prehistoric samples (from the Neolithic to the Iron Age) and 166 historic samples (from Antiquity to the Middle Ages), based on their respective archaeological and chronological contexts (Supplementary Table S2). Although the newly analyzed samples are heterogeneous and originate from different historical periods, their genetic signatures conform to the broader patterns expected for the region.

**Figure 4:**
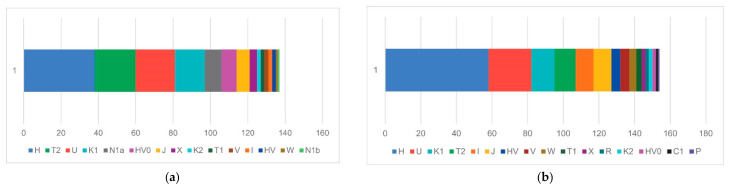
mtDNA haplogroup diversity in prehistoric (**a**) and historic (**b**) Croatia.

**Figure 5:**
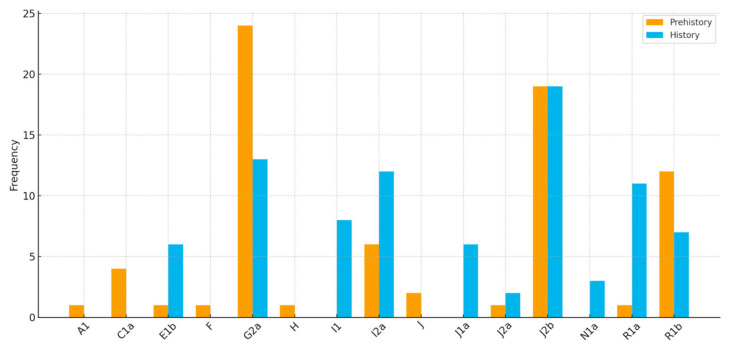
A comparison of prehistoric (yellow) and historic (blue) ancient mtDNA haplogroup profiles.

**Figure 6:**
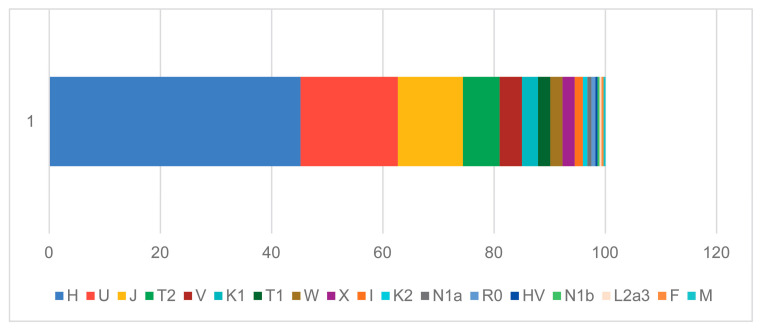
mtDNA haplogroup diversity in modern Croatia.

The prehistoric mtDNA profile of the Croatian region shows a balanced mixture of typically European maternal lineages. Haplogroups H, T2, U, and K1 appear at similar frequencies, forming the core of the prehistoric dataset (Figure 4 and Figure 5). These are complemented by substantial contributions from other haplogroups (e.g., N1a, HV0, J, X, K2, T1, V, W, and I), making a heterogeneous genetic landscape. This pattern reflects the composite ancestry of early populations in the region, combining Mesolithic hunter-gatherer lineages (notably U) with the diverse maternal haplogroups introduced by early Neolithic farmers (K1, T2, J, HV, and the characteristically Neolithic N1a). In contrast, the historic mtDNA profile shows a significant restructuring of maternal ancestry. Haplogroup H greatly expands and becomes the dominant lineage, occupying roughly half of the entire maternal gene pool. Other prehistoric lineages—U, K, T, J, HV, and I—remain present and in some cases increase, demonstrating strong genetic persistence with earlier populations. However, the distinctly Neolithic N1a lineage disappears entirely, mirroring its well-documented decline across Europe after the Early Neolithic [52]. Several new mitochondrial haplogroups appear in low frequencies, such as R, C1, and P, indicating more pronounced population movements and expansion of maternal diversity during the Roman and medieval periods. The modern Croatian mtDNA pool (Figure 6) aligns with the contemporary European genetic landscape. It represents a continuation of the observed historical patterns, primarily reflected in the steady increase of the H clade. Haplogroup H accounts for 45.2% of the modern gene pool and remains the dominant maternal lineage, followed by U, J, T2, V, K1, and other low-frequency haplogroups. N1a is present but at a low frequency, and other minor haplogroups, such as N1b, R0, L2a3, F, and M, were also introduced into the maternal gene pool during later historical periods [49,53].

**Figure 7:**
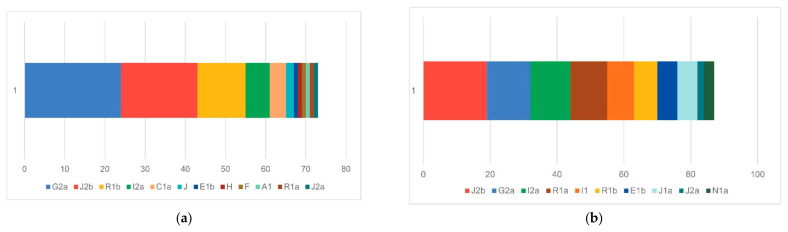
Y haplogroup diversity in prehistoric (**a**) and historic (**b**) Croatia.

**Figure 8:**
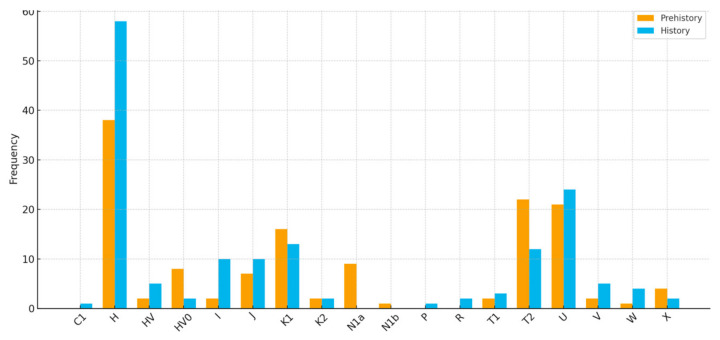
A comparison of prehistoric (yellow) and historic (blue) ancient Y-chromosome haplogroup profiles.

From the paternal perspective, Croatian ancient samples are characterized by two main genetic layers evident in the Y-chromosome haplogroup composition (Figure 7 and Figure 8). The prehistoric Y-chromosome profile is dominated by the Neolithic G2a and J2b markers, with moderate levels of Bronze Age R1b and Paleolithic I2a, and small contributions from rare Paleolithic lineages, including C1a, E1b, H, F, and A1. In contrast, the historic dataset shows a marked rise in R1a, I2a, I1, and E1b, while G2a declines and J2b takes the lead in terms of haplogroup dominance in historic times. Several prehistoric lineages disappear entirely (C1a, H, and F), and new lineages appear in the historic period (N1a), indicating specific shifts in the paternal ancestry composition.

**Figure 9:**
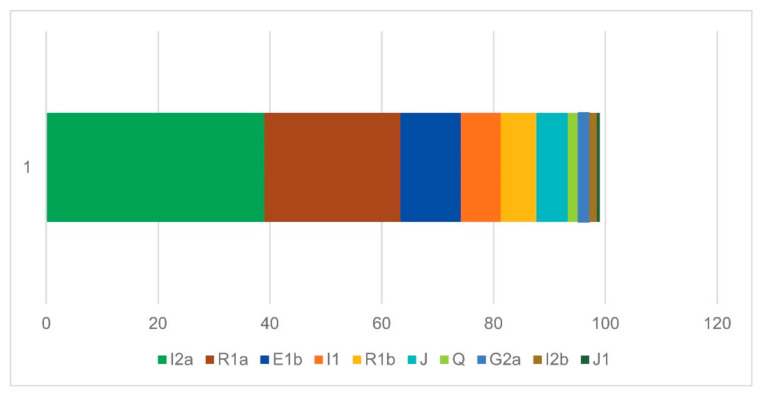
Y-chromosome haplogroup diversity in modern Croatia.

When compared to the modern dataset (Figure 9), a substantial increase in I2a is evident, rising from a low prehistoric presence to a moderate historic level, and ultimately becoming the dominant modern lineage at 39%. R1a also grows substantially, more than doubling its representation from a scarce presence in prehistory to a moderate historic level, and becoming the second most prevalent haplogroup in Croatia today. E1b exhibits a similar pattern to R1a, albeit to a lesser extent, while I1 remains present at similar proportions, indicating moderate stability over time. In contrast, several prehistorically important haplogroups (especially G2a and the J sublineages) decline sharply in the modern population, and today they are at low but stable levels. It is also evident that the steppe-related R1b clade has decreased in frequency over time, from being the third most prevalent prehistoric lineage to having a relatively low representation today. We can conclude from this observation that a general decrease in Neolithic and Bronze Age signals is evident over time, allowing for more space for the influences of the more recent population in Antiquity and the Middle Ages.

## 4. Discussion

### 4.1. Mitochondrial and Y-Chromosomal Diversity in (Pre)historic Dalmatian Populations

The mitochondrial haplogroup composition of the newly analyzed individuals reflects a typical European maternal ancestry, dominated by haplogroups H, U, HV0, K1, and T. Haplogroup H, the most frequent in our dataset, aligns with its known prevalence in European populations since the Neolithic transition [38,54]. The occurrence of U subclades (U2d2, U3a1, and U4a2) suggests deep local ancestry rooted in European hunter-gatherer populations, yet with connections to Near Eastern and Caucasian gene pools, consistent with earlier findings from the SEE [4,9].

The detection of HV0 and K1a, which likely originated in the Near East, reinforces the importance of this region as a source of maternal genetic input into the Adriatic during the Neolithic and Bronze Age. The presence of rare haplogroups such as N1b and I1a further attests to low-frequency but detectable genetic diversity, possibly introduced through episodic long-distance interactions along Mediterranean maritime routes. Taken together, these results suggest that the maternal gene pool of (pre)historic Dalmatia was shaped by long-term regional persistence interwoven with low-level gene flow from surrounding regions, mirroring the patterns observed in other parts of SEE [3].

Y-chromosomal data of newly sequenced samples suggest high paternal heterogeneity, indicating multiple demographic strata and a pronounced male-mediated gene flow. The dominance of Neolithic-derived haplogroups J1a, J2a, and J2b points to a strong and persistent signature of Neolithic ancestry. Haplogroup J has long been associated with the spread of early farming communities into southeastern Europe (SEE), and its continued presence throughout the Bronze Age and Antiquity underscores stable genetic links between the Adriatic, the Near East, and the broader Mediterranean world [3,4,11]. The simultaneous detection of both J2a and J2b further supports this narrative, as these subbranches are characteristic markers of Neolithic settlement and later regional interaction within SEE. The occurrence of R1a in the prehistoric part of the sample confirms that R1a was already present in SEE, albeit at low frequencies, while its appearance in the early historic individual aligns with genomic evidence suggesting increased prevalence during the early medieval period, likely influenced by demographic movements from Central and Eastern Europe, including those linked to Slavic expansions [2,5,6,10]. Haplogroup R1b, associated with the substantial Bronze Age influx of steppe-related ancestry accompanying the spread of Indo-European-speaking populations into Europe, was detected in only a single prehistoric individual. Given that most of the analyzed individuals date to the Bronze and Iron Ages, the scarcity of R1b is unexpected and may reflect local demographic variability and complex patterns of Bronze Age mobility in the eastern Adriatic [9,15,50].

Although absent from the newly analyzed sample, the I2a haplogroup has been present in this region for the past several millennia. Namely, the wider Croatian ancient DNA database (Supplementary Table S2), which spans the Neolithic to the Middle Ages and includes 165 male samples, confirms its presence in the SEE in all (pre)historic periods. Previous studies have also suggested its Paleolithic SEE origin [2,10] and Mesolithic presence [9]. This disputes the thesis that this haplogroup reached the region exclusively during medieval migrations, although our data suggests that its initial frequencies were significantly lower compared to today’s genetic composition.

The presence of haplogroup A1, which is primarily observed in African populations, is rare in Europe but has been sporadically reported in other Mediterranean contexts. Isolated occurrences in ancient Levantine and southern European samples have been noted in the literature, and these have been tentatively attributed to individual-scale mobility rather than to sustained gene flow or broader demographic processes [10].

### 4.2. Patterns of Continuity and Change in Croatian Maternal and Paternal Lineages over Time

When integrated with previous genomic and archeological evidence, our results support a model of partial genetic persistence with visible migration waves. The general prehistoric mtDNA profile of modern-day Croatia suggests continuity in major maternal lineages alongside specific minor shifts across time. The prehistoric pattern supports the hypothesis that a population was shaped by early Neolithic farming expansions, mixed with enduring Mesolithic European ancestry. The historic rise in H, U, T, J, K, and other less pronounced lineages suggests ongoing integration of pan-European and possibly steppe-related maternal lines. At the same time, the decline in N1a frequency aligns with its known decrease after the Neolithic period. It reappears in the modern Croatian population, albeit at a low frequency, as is the case in the general European population. The presence of new and rare lineages in the historic sample can be interpreted as a sign of expanded long-distance contact and increased female-mediated mobility during later periods, likely driven by trade, migration, and cultural exchange. The modern Croatian gene pool exhibits relatively high mtDNA haplogroup diversity, dominated by West Eurasian haplogroups H, U, J, T, V, and K, followed by W, X, and I. Other low-frequency haplogroups also show similarities with prehistoric populations [53,55,56].

The general prehistoric Y-chromosome profile of modern-day Croatia suggests a substantial transformation of male-line ancestry between the prehistoric and historic periods. The prehistoric dominance of G2a and J2b suggests a prominent early farmer influence, while a low but evident presence of I2a indicates deep local Mesolithic continuity, as highlighted previously [2,10]. In contrast, the historic profile shows signs of demographic transformation: substantial new inputs from R1a (linked to Slavic and broader Indo-European expansions), as well as from I1 (Northern European/Germanic) and J1a (Near Eastern/Arabian), along with rising E1b (southern European) and new N1a signals, which indicates increased mobility and cultural interactions during the Iron Age, Roman/Byzantine periods, and medieval migrations. With time, G2a declines, J2b remains strong, and I2a grows, suggesting both continuity and assimilation. Overall, the data points to a shift from a primarily early farming and local hunter-gatherer paternal landscape to a more diverse and mobile historic population shaped by multiple migration waves.

The observed asymmetry between the maternal and paternal perspectives suggests long-standing patrilocal and patrilineal social structures, in which women moved between communities more frequently than men, thereby preserving mtDNA diversity while making Y chromosomes vulnerable to changes in haplogroup frequency [57]. Additionally, the effective population size of the Y chromosome is lower than that of mtDNA, making Y lineages more sensitive to genetic drift and bottlenecks, especially during periods of male-biased conflict or elite dominance [58]. As a result, while mtDNA often shows similar patterns from prehistory to the present, Y-DNA reflects punctuated episodes of male-driven demographic expansion and social transformation.

When compared with modern Croatian genetic diversity, a significant increase in I2a frequency is observed, making it the dominant paternal lineage today [10]. Our research on ancient DNA genomes from Croatia aligns with our previously published work, which shows that although the presence of the I2a haplogroup during prehistory was limited, it remains clearly present across all time periods and is likely to have originated in the Paleolithic SEE [6]. This outcome is somewhat in contrast with modern genetic data, where this is by far the most frequent haplogroup in modern Croatia and neighboring SEE countries [10,59]. While the ancient dataset is still relatively small and unevenly distributed across time periods, it nevertheless highlights trends that may differ from widely held assumptions about how the paternal gene pool in Southeastern Europe was formed.

In conclusion, this work introduces a robust, uniparental-focused framework for reconstructing long-term population dynamics in SEE by jointly analyzing newly-generated and published ancient DNA from Croatia. The integration of maternal and paternal lineages across multiple prehistoric and historic periods and locations provides novel insight into sex-biased mobility at a regional scale.

#### Limitations of the Study

It is essential to acknowledge the potential influence of sampling bias arising from the relatively small sample size of this study. For this reason, the newly generated data were integrated into a larger dataset of previously published ancient genomes, enabling more robust inferences at the population level. Uniparental markers provide lineage-specific resolution but reflect only a small fraction of the genome and are highly sensitive to drift and sex-biased processes. Accordingly, conclusions drawn from mtDNA and Y-chromosome data should be interpreted as complementary to, rather than substitutes for, genome-wide analyses. The dataset is also somewhat unevenly distributed across archaeological periods and geographic regions—specific time intervals and contexts are less well represented than others, due to limited access to ancient human remains. In addition, genetic data from present-day Croatia are primarily derived from samples collected for population genetic studies, whereas ancient DNA datasets originate from archaeological contexts, reflecting different sampling frameworks. Taken together, these results should therefore be viewed as indicative rather than exhaustive, highlighting the need for increased temporal and spatial sampling in future studies.

## Data Availability

All data is provided in the manuscript or the Supplementary Materials.

## Supplementary Materials

The following supporting information can be downloaded at: https://www.mdpi.com/article/10.3390/genes17010080/s1. References [60,61,62,63,64,65] are cited in the Supplementary Materials.
